# Correction to “A Multi‐Taxa Approach to Estuarine Biomonitoring: Assessing Vertebrate Biodiversity and Ecological Continuity Using Environmental DNA Metabarcoding in the Rance River (Brittany, France)”

**DOI:** 10.1002/ece3.73546

**Published:** 2026-04-20

Haderlé, R., A. Carpentier, G. Kervarec, et al. 2026. “A Multi‐Taxa Approach to Estuarine Biomonitoring: Assessing Vertebrate Biodiversity and Ecological Continuity Using Environmental DNA Metabarcoding in the Rance River (Brittany, France).” *Ecology and Evolution* 16, no. 3: e73237. https://doi.org/10.1002/ece3.73237.

In the originally published version of this article, the authors’ names were presented incorrectly, with given names and family names reversed. The correct author list is:

Rachel Haderlé, Alexandre Carpentier, Gaël Kervarec, Anne Lizé, Nils Teichert, Visotheary Ung and Jean‐Luc Jung.

The online version of this article has been corrected accordingly.

We apologize for this error.

