# Supplementary material for: A Multi‐Taxa Approach to Estuarine Biomonitoring: Assessing Vertebrate Biodiversity and Ecological Continuity Using Environmental DNA Metabarcoding in the Rance River (Brittany, France)
Source: Ecol Evol. 2026 Mar 9;16(3):e73237. doi: 10.1002/ece3.73237 (PMC12971189; doi:10.1002/ece3.73237)
Supplement: Supplementary file 2 — Appendix S2: ece373237‐sup‐0002‐AppendixS2.docx. [file ECE3-16-e73237-s001.docx]

**Appendix S2** "P-values from pairwise permutation tests comparing methods across different biodiversity metrics.

| **Indicator** | **Station1** | **Station2** | **p_value** |
| --- | --- | --- | --- |
| **Taxonomic richness** | ANACONOR | DCE_rance | 1 |
| **Taxonomic richness** | eDNA | DCE_rance | 0.343 |
| **Taxonomic richness** | DCE_rance | ANACONOR | 1 |
| **Taxonomic richness** | eDNA | ANACONOR | 0.678 |
| **Taxonomic richness** | DCE_rance | eDNA | 0.33 |
| **Taxonomic richness** | ANACONOR | eDNA | 0.678 |
| **FRic** | ANACONOR | DCE_rance | 0.321 |
| **FRic** | eDNA | DCE_rance | 1 |
| **FRic** | DCE_rance | ANACONOR | 0.36 |
| **FRic** | eDNA | ANACONOR | 0.656 |
| **FRic** | DCE_rance | eDNA | 1 |
| **FRic** | ANACONOR | eDNA | 0.644 |
| **SES.FRic** | ANACONOR | DCE_rance | 0.313 |
| **SES.FRic** | eDNA | DCE_rance | 1 |
| **SES.FRic** | DCE_rance | ANACONOR | 0.374 |
| **SES.FRic** | eDNA | ANACONOR | 0.666 |
| **SES.FRic** | DCE_rance | eDNA | 1 |
| **SES.FRic** | ANACONOR | eDNA | 0.636 |
| **PD** | ANACONOR | DCE_rance | 1 |
| **PD** | eDNA | DCE_rance | 0.706 |
| **PD** | DCE_rance | ANACONOR | 1 |
| **PD** | eDNA | ANACONOR | 0.332 |
| **PD** | DCE_rance | eDNA | 0.688 |
| **PD** | ANACONOR | eDNA | 0.333 |
